# Triple-Drug Treatment Is Effective for Lymphatic Filariasis Microfilaria Clearance in Samoa

**DOI:** 10.3390/tropicalmed6020044

**Published:** 2021-04-01

**Authors:** Patricia M. Graves, Sarah Sheridan, Jessica Scott, Filipina Amosa-Lei Sam, Take Naseri, Robert Thomsen, Christopher L. King, Colleen L. Lau

**Affiliations:** 1 College of Public Health, Medical and Veterinary Sciences, James Cook University, Cairns, QLD 4870 and Townsville, QLD 4811, Australia; Jessica.Scott2@my.jcu.edu.au; 2 School of Public Health and Community Medicine, University of New South Wales, Sydney NSW 2033, Australia; sarah.sheridan1@health.nsw.gov.au; 3 School of Medicine, National University of Samoa, Apia, Samoa; F.amosa@nus.edu.ws; 4 Ministry of Health, Apia, Samoa; taken@health.gov.ws (T.N.); robertt@health.gov.ws (R.T.); 5 Center for Global Health, School of Medicine and Veterans Affairs Administration, Case Western Reserve University, Cleveland, OH 44106, USA; cxk21@case.edu; 6 School of Public Health, The University of Queensland, Brisbane, QLD 4006, Australia; colleen.lau@uq.edu.au; 7 Research School of Population Health, The Australian National University, Canberra, ACT 2601, Australia

**Keywords:** lymphatic filariasis, Samoa, microfilaria, DEC, albendazole, ivermectin

## Abstract

Following the first triple-drug mass drug administration (MDA) for lymphatic filariasis in Samoa in 2018, unexpected persistence of microfilaria (Mf) positivity in 18 (15%) of 121 antigen-positive persons was observed in a nationwide household survey 1–2 months later. Of the 18 Mf positive persons, 14 reported taking the MDA, raising concerns about MDA efficacy. In 2019, 5–6 months after the 2018 survey, a monitored treatment study was done to evaluate directly observed weight-based treatment in these Mf positive individuals. Mf presence and density were assessed before and 7 days after treatment, using 1 mL membrane filtered venous blood, and 60 uL thick blood films on slides prepared from venous or fingerprick blood. All 14 participants were still Mf positive on filters from venous blood pre-treatment samples, but two were negative by slide made from the same samples. Mf were cleared completely by day 7 in 12 of 13 participants followed up, and by day 30 in the remaining participant. Filtered blood using EDTA samples (to reduce clumping of Mf) is preferred over slides alone for improving the likelihood of detecting Mf and estimating their density. The triple-drug MDA strategy was effective at clearing Mf when given and taken at the correct dose.

## 1. Introduction

Lymphatic filariasis (LF) is a helminth worm transmitted by mosquitoes. The adult worms live in the lymphatic system, potentially causing chronic disability due to limb and scrotal swelling. A mated pair of adult worms lives for 5 to 7 years and can produce millions of microfilariae (Mf) that circulate in peripheral blood. The number of LF infected persons worldwide in 2000 is usually estimated to have been 130 million [1], although a revised update recently generated using new modelling methods using surveys from 1990 to 2018 suggested that as many as 199 million people may have been infected in 2000 [2]. The Global Programme to Eliminate Lymphatic Filariasis (GPELF) has conducted an intensive mass drug administration (MDA) programme since 2000 to interrupt transmission of LF [3], and the burden of infection is estimated to have been reduced dramatically to 50 to 70 million infections in 2018 [1,2]. By 2020, 7.7 billion MDA treatments had been delivered and 17 countries or territories (eight of which are in the Pacific region [4]) have been validated as having eliminated LF as a public health problem [3,5].

However, some countries are experiencing challenges including stagnation of progress, delay in MDA initiation, persistence of transmission hotspots, failing Transmission Assessment Surveys (TAS) during post-MDA surveillance, and resurgence (increasing LF antigen prevalence) after MDA had stopped [6,7,8,9]. These countries include Samoa and American Samoa which have efficient day-biting *Aedes polynesiensis* and other vectors, including night biting species, with exposure 24 h a day and few options for vector control. It is well recognized that *Aedes* vectors are more efficient than other genera, and this is reflected in the lower WHO survey thresholds in *Aedes* transmission areas. For those reasons, in 2017 the GPELF recommended introduction of triple-drug MDA using ivermectin, diethylcarbamazine (DEC), and albendazole (IDA) to address some of these obstacles [10]. The triple-drug regimen is expected to rapidly clear all Mf within one week of treatment [11] and partially sterilize the adult worms, although Mf may reappear in a small proportion of treated people one or two years later [12,13].

The Pacific region has a long history of high LF prevalence and MDA interventions, and Samoa has done 20 rounds of MDA overall since 1965. Before GPELF, Samoa conducted eight rounds of MDA with DEC including weekly and/or monthly doses for 12–18 months in 1965 and 1971 followed by annual doses in 1982, 1983, 1986 and 1993–1995 inclusive, and two rounds of DEC plus ivermectin in 1996–1997 [14]. Samoa was then the first country to initiate MDA with DEC and albendazole in 1999 at the beginning of the Pacific Programme to Eliminate LF (PacELF) before the official start of GPELF in 2000 [15]. Under PacELF, Samoa conducted five nationwide MDA rounds between 1999 and 2003, but did not achieve the recommended threshold in a community cluster survey of all ages in 2004 [16], so a further three nationwide rounds of two-drug MDA were distributed in 2006, 2008 and 2011. After failing a TAS in one evaluation unit in 2013, two further targeted rounds of MDA were distributed in that area only in 2015 and 2017, but all three evaluation units in Samoa failed TAS-2 later in 2017. Due to apparently resurging antigen prevalence back to 1999 levels, the country initiated triple-drug MDA with the first nationwide distribution successfully completed in August 2018 [17].

A large population representative LF community survey as part of the Surveillance and Monitoring for Elimination of LF and Scabies in Samoa (SaMELFS) project was conducted in Oct-Nov 2018, 8–11 weeks after the August 2018 MDA, and showed age- and gender-standardized antigen prevalence of 4.0% (95% CI 2.8–5.6%) in 3940 participants aged 5 years and over [9]. The MDA coverage was reported to be very good with 80.2% of the total population reporting taking MDA [17]. Amongst the 122 Ag-positive participants, slides were available for 121 (99.2%) [9], and 18 (14.9%) were Mf-positive. Surprisingly, 14 of the 18 Mf-positive persons reported taking the IDA pills in 2018, raising concerns about the effectiveness of IDA in Samoa. The estimated post-MDA Mf prevalence of 0.6% (0.3–1.0%) nationwide and 1.7% (0.7–4.1%) in known hotspots [9] also caused concern that IDA might not be as effective as expected in Samoa.

This “monitored treatment” study is a preliminary investigation into the reason(s) for persistent Mf in Ag-positive people, the majority of whom reported taking IDA in Samoa in 2018. The primary objective of the study was to assess the effectiveness of appropriately dosed IDA in clearing Mf from Mf-positive people identified in the October–November 2018 survey. We revisited these Mf-positive persons in March–April 2019, i.e., 5–6 months after the previous survey. This study aims to exclude three hypotheses about the potential reasons for Mf persistence: (i) inadequate dosage due to incorrect weighing or administration of pills; (ii) incorrect reporting about participation, i.e., non-compliance or partial compliance in taking pills; and (iii) drug resistance in the local filarial worm population.

## 2. Materials and Methods

### 2.1. Study Setting

Samoa is an independent country in the South Pacific (population ~200,000 in 2018), consisting of two main tropical islands, Upolu and Savai’i, and a number of sparsely populated smaller adjacent islands. Samoa is divided into four administrative regions, with ~19% of the population living in Apia Urban Area (AUA), 35% in Northwest Upolu (NWU), 23% in Rest of Upolu (ROU), and 22% in Savai’i (SAV) [18]. The larger islands are made up of tropical forests, mountains, valleys, wetlands, and fringing reefs, and the majority of the population live in small coastal villages in a rural setting.

### 2.2. Study Design

We revisited Mf-positive persons in March–April 2019, i.e., 5–6 months after the 2018 survey, to provide treatment with one dose of IDA. We assessed the effectiveness of IDA in clearing Mf by comparing Mf presence and density at baseline (pre-treatment) and after directly observed treatment using recommended weight-based dosages of all three medications. We collected blood samples at baseline, and at 3 h and 7 days after treatment. The plasma sample at 3 h post-treatment was collected to allow for pharmacokinetic studies (plasma concentrations of ivermectin, DEC and albendazole) to determine whether the recommended dosages were sufficient for achieving effective plasma concentrations. If Mf were not cleared by 7 days post-treatment, repeat samples were collected at 30 days post treatment.

### 2.3. Ethics Approval and Consent

Ethics approvals were granted by the Samoa Ministry of Health and The Australian National University Human Research Ethics Committee (protocol 2018/341). The study was conducted in close collaboration with the Samoa Ministry of Health, the WHO country office in Samoa, and the Samoa Red Cross. All participants or guardians gave written informed consent.

### 2.4. Treatment of Mf-Positive Persons

Each person was weighed by a doctor or nurse using medical-grade scales. Treatment with ivermectin (150–200 ug/kg, Merck), DEC (6 mg/kg, Eisai), and albendazole (one 400 mg tablet, GSK) was provided according to weight as per the Samoa Ministry of Health 2018 MDA schedule [17] shown in Table 1. Medications were donated for the triple drug MDA in 2018 by Merck, Eisai and GSK and provided through WHO. Treatment was directly observed by study team members.

### 2.5. Blood Samples and Laboratory Methods

Detection of filarial Mf on filtered blood was first described by Wylie in 1970 [19] for veterinary parasites, with modified methods proposed by Chularerk and Desowitz in 1970 [20] and by Dickerson et al. for polycarbonate filters in 1990 [21]. The method has been used extensively in the Pacific region including in Tonga [22], Fiji [23], Samoa [23] and PNG [24], including most recently in clinical trials of safety and efficacy of the triple-drug MDA regime [11]. Previous studies have usually used blood with EDTA or citrate as anticoagulant, although heparin with Teepol detergent was used in one study in Tonga [20].

A recent systematic review attempted to standardize the diagnostic methods used for assessing Mf prevalence in blood samples collected from participants [25]. The methods evaluated were counting chamber (>50 uL blood); membrane filtration (1 mL blood); Knotts technique (1 mL); and slides made with ≥40 uL of blood. Estimated Mf prevalence in the persons concurrently sampled was 2.39 (95% CI 1.62–3.53) times higher when using membrane filtration and 1.37 (95%CI 0.81–2.30) times higher when using slides with ≥40 uL blood [25], compared to 20 uL blood slides. Previous studies in Haiti with *W. bancrofti* have observed higher Mf densities on slides (prepared using 20 uL fingerprick capillary whole blood) than in concurrent filtered venous samples [26,27]. Therefore, we decided also to evaluate slides made from venous and fingerprick blood in order to investigate the validity of this finding and maximize the sensitivity of the study for detecting Mf clearance.

Based on the above findings from previous studies, we used both membrane filters and 60 uL three-line slides to evaluate Mf presence and density. Plasma samples were prepared from the venous blood and stored for future pharmacokinetic studies if required. After consulting recent users of the membrane filtration technique, we chose initially to use heparinized blood samples. Although heparin is known to cause clumping of leucocytes and Mf in blood samples [28], its advantage is that the same blood sample can be used for the Alere Filariasis Test Strip (FTS) antigen test, for which EDTA or citrate blood is not recommended. We decided to also test venous samples in EDTA for practical reasons that evolved as the study progressed.

Samples taken from study participants included venous blood in 10 mL lithium heparin and EDTA Vacutainers (BD, Macquarie Park, NSW, Australia) and finger-prick blood (using contact-activated spring-loaded high blood flow 1.5 × 2 mm lancets BD cat 366594) in lithium heparin Microtainers (BD cat 365965). Blood samples were collected in both heparin and EDTA where possible, as well as fingerprick samples in heparin Microtainers for the FTS tests and to compare Mf counts from venous and capillary blood.

With heparin, clumping was very marked and occurred at both macro and micro levels (Supplementary Figure S1), making counting on high density Mf samples very difficult (Supplementary Figure S2). The clumping was minimized with EDTA, making the filters much clearer and Mf easier to count. Therefore, we collected blood in both heparin and EDTA tubes for the filters where possible after the first 10 participants, and took both specimen types (plus slides) into account in determining Mf presence.

Household members of the study participants were also interviewed about MDA participation in 2018, and with their consent, were tested by FTS on fingerprick blood samples. If Ag-positive, slides were prepared, stained and examined for Mf in Samoa.

All blood was kept cool in insulated containers during transport to the field laboratory. A flow chart illustrating the schedule of collections is given in Table 2 and summarized below:

- Venous blood samples in heparin or EDTA were used on the same day of collection or (rarely) stored at 4 °C until the next morning. They were used for:
  (a) membrane filters;
  (b) three-line thick blood films on slides (60 uL);
  (c) collection of plasma (for potential pharmacokinetic assays) by centrifuging the remaining sample at >1000× *g* for 10 min.
- Fingerprick blood samples were used on the day of collection or the next day after storage at 4 °C to:
  (a) test for circulating filarial antigen (Ag) using Alere Filariasis Test Strips (FTS);
  (b) prepare dried blood spots on TropBio filter papers (Cellabs) for future testing for anti-filarial antibodies (Ab);
  (c) prepare three-line thick blood films on slides (60 uL per slide).

### 2.6. Procedure for Membrane Filters

The venous blood samples were handled and prepared for Mf detection using procedures derived from previous studies [11,21]. Briefly, we used Millipore Isopore 25 mm polycarbonate filter discs and Swinnex EMD 25 mm filter holders (Merck, Brisbane, QLD, Australia). After assembling the filter, 10 mL of distilled or bottled water was passed through the filter assembly using a 10 mL syringe. Venous blood samples in Vacutainers were mixed thoroughly by inverting four to five times, then 1 mL of blood was drawn up into a 1 mL syringe using a 20 G needle and gently passed through the filter. Immediately afterwards, two lots of 10 mL of distilled or bottled water were passed through the filter to lyse the red blood cells, followed by 10 mL of air to dry the filter. The filter was removed from the assembly using tweezers, and placed face up on a microscope slide to dry before staining with Giemsa.

Second filters were collected for potential future sequencing of Mf from each venous sample where possible. The procedure was the same as preparing filters for staining except that after lysing the blood with water, 1 × 1 mL of RNALater solution (Thermo Fisher, Townsville, QLD, Australia) was passed through the filter, followed by 10 mL of air. Filters were placed using tweezers into Eppendorf tubes which were filled to the brim with RNALater and stored at −70 °C.

Filters were allowed to dry at least overnight (protected from ants) before staining or storage in slide boxes. All filters were shipped to Australia for counting.

### 2.7. Filariasis Test Strips

The Alere Filariasis Test Strip (FTS) (Scarborough, ME, USA) was used to detect circulating filarial Ag. Using a micropipette, 75 μL of heparinized blood was placed onto the FTS, and assessed after 10 min per manufacturer’s instructions. If sufficient blood was available, positive tests were repeated to confirm the result.

### 2.8. Blood Slides

For all blood samples with a positive FTS result, thick blood slides were prepared according to WHO guidelines as previously described [9]. Three 20 μL lines of blood were placed onto a single slide using a micropipette, and up to three slides were prepared if sufficient blood was available. Slides were dried flat for one day and then in boxes for 2 more days, protected from ants. They were dehaemoglobinised in water for 15 min, carefully removed, dried again and stored in slide boxes. One slide from each participant was stained and examined immediately; the remainder of the slides were stored and shipped to Australia before staining.

### 2.9. Staining of Filters and Slides

Dehaemoglobinised slides were fixed in 100% methanol for 3 min before staining. Filters and slides were stained with 2% Giemsa (VWR Giemsa Stain Improved R66 Gurr; Bio-Strategy Pty Limited, Campbellfield, VIC Australia) in distilled water for 50 min, rinsed in distilled water, allowed to dry and examined at 100× and 400× magnification.

All slides and filters were read independently and blindly by two readers (PG and JS) and the results reported are the average counts. The numbers of Mf on the whole filter or slide were counted. Counts from 60 uL slides were extrapolated to equivalent counts per mL.

### 2.10. Statistical Analysis

Mf counts were highly skewed and were transformed to logs for statistical comparisons; 1 was added to all counts/mL to allow for 0 counts in log transformations and plots. Log (Mf/mL + 1) counts in heparinised blood for different sample types (venous and fingerprick) and specimens (filter or slide) at day 0 were compared using one-way ANOVA with non-independent samples. Median Mf and median log (Mf/mL + 1) counts at day 0 were compared using non-parametric K-sample tests and Wilcoxon matched pairs signed rank test. Pearson’s correlation coefficient was estimated for log ((Mf/mL + 1) counts from individuals using different sample and specimen types. Analysis was done using STATA 16.

## 3. Results

### 3.1. Study Participants

Of the 18 Mf-positive people identified in 2018 SaMELFS survey, 17 gave information in 2019 (in one case by telephone) about MDA participation, and 14 participants were enrolled in the monitored treatment study involving blood sampling. Four people did not enrol in the monitored treatment and follow-up due to declining to participate (*n* = 1), being overseas (*n* = 1), ineligible due to breastfeeding (*n* = 1) and being unable to schedule a visit (*n* = 1). The 14 full participants lived in 12 different villages (10 in Northwest Upolu (NWU), one in Rest of Upolu, and one in Savai’i). No Mf positive persons were identified in 2018 in Apia Urban Area. The villages of Vaiusu and Leauva’a (both NWU) had two Mf positive participants, one in each of two separate households; the other villages each had one Mf positive participant. Their ages ranged from 5 to 74 years, and there were 8 females and 6 males (Table 3). The range of weights of adult participants (aged 20 to 74) was 67 to 110 kg, with total numbers of tablets given in 2019 ranging from 9 to 15 according to Table 1. The one 5 year old child weighed 18 kg and was given one tablet each of ivermectin, DEC and albendazole.

### 3.2. MDA Participation in 2018

At the time of the 2018 survey, each person or their parent/guardian was asked if they were aware of the MDA, eligible for MDA, and whether they were offered it. Then, they were asked if they took all the pills, and if not, why not. Results for the whole survey were presented by Willis et al. [17], and for the current study participants in Table 3. When 17 of the 18 Mf positive persons from 2018 were interviewed and probed in more depth in the current study in 2019 about participation in the 2018 MDA, some of their responses in 2019 differed from their previous reports made at the time of the 2018 survey (Table 3).

In 2018, 14 of the 18 participants answered Yes about MDA participation. In 2019, five of these (3 in the full follow-up group) changed their answers to No, and one other reported that they had not taken all three drugs (Table 3 and Table 4). Reported full compliance in Mf positive persons was thus 77.8% (*n* = 18) when asked in 2018 and 41.2% (*n* = 17) when asked in 2019. We have no way of verifying the self-reported compliance in 2018 or number of pills actually taken, but anecdotally heard that some participants were not weighed at that time.

Of the 52 family members who participated in 2019, 27 family members responded in both the 2018 and 2019 surveys. (Table 4). One family member changed their answer from Yes to No, while another changed in the opposite direction. No other family members changed their report of MDA compliance, which was thus reported as 88.9% (*n* = 27) in both years’ surveys.

### 3.3. Antigen and Mf Results at Baseline in 2019

**Figure 1:**
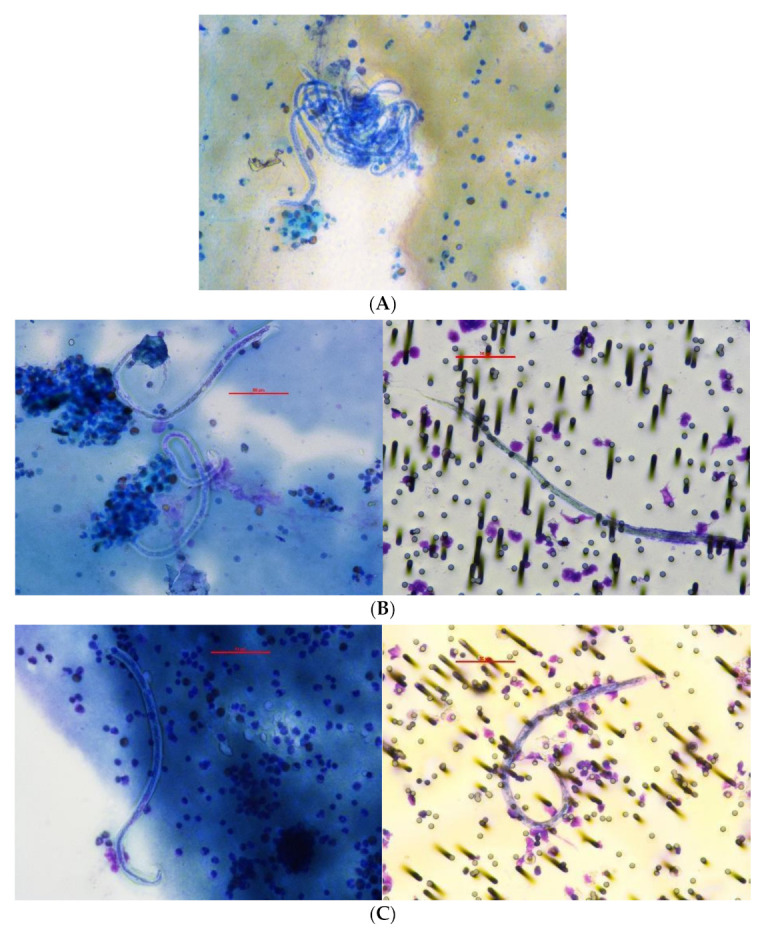
Participant ID 1: Microfilariae on slides and filter of 59 year old male in 2018 and 2019 (day 0 and 7): (**A**) 2018 slide from fingerprick heparin blood, 400×; (**B**) 2019 day 0 slide from heparin fingerprick blood and filter from venous EDTA blood, 400×; (**C**) 2019 day 7 slide from heparin venous blood and filter from venous EDTA blood, 400×.

All 14 Mf positive participants were Ag-positive by FTS at visit 1 (pre-treatment) and had Mf detected on at least one of their pre-treatment blood samples. Examples of Mf observed in Participant 1 are shown in Figure 1.

Among the 52 family members tested by FTS in 2019, 11 (21.2%) were Ag-positive, while among the 27 of these family members who participated in both 2018 and 2019 surveys, five (18.5%) were Ag-positive (Table 4). None of the Ag-positive family members was Mf-positive on either of two 60 uL slides in 2019. All Ag-positive family members were treated.

### 3.4. Mf Counts in Study Participants Before and After Treatment

**Figure 2:**
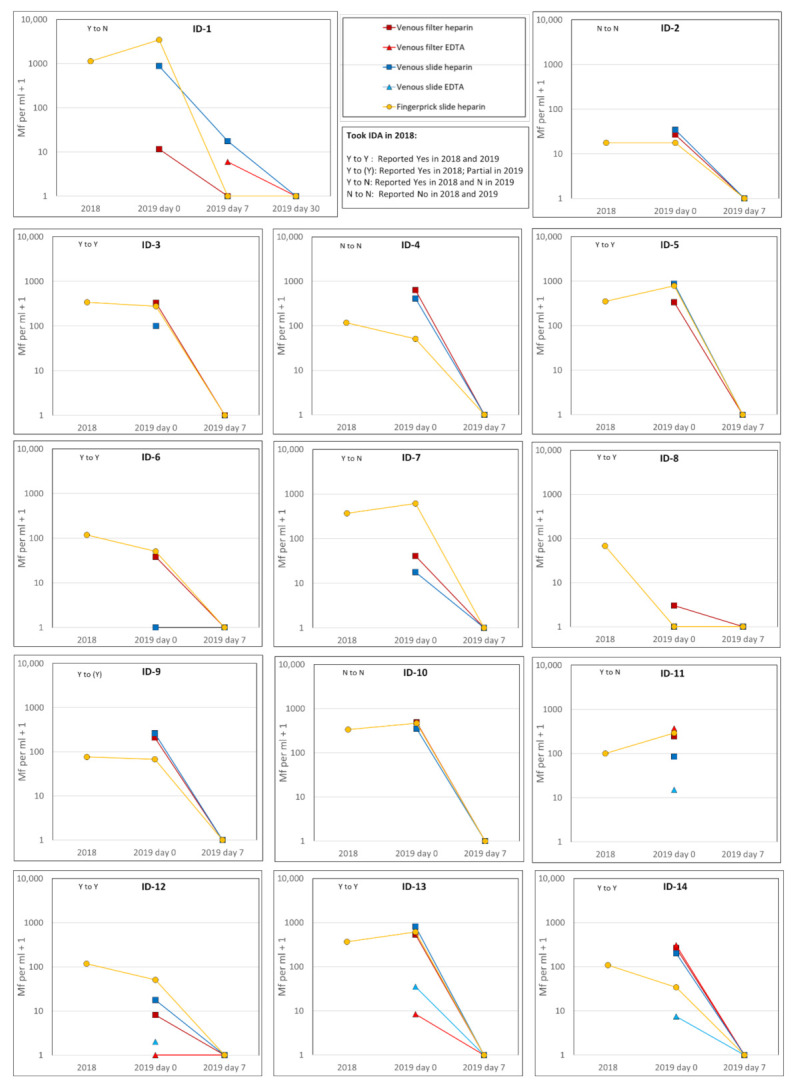
Mf counts per mL + 1 in blood samples taken in 2018 and 2019. Red squares: venous heparin filter; red triangles: venous EDTA filter; blue squares: venous heparin slide; blue triangles: venous EDTA slide; yellow circles: fingerprick heparin slide. Note for ID-11: withdrew after 2019 day 0 (after treatment). Note for ID-12: venous draw failed at 2019 day 0; 0.7 mL of heparin fingerprick blood used for heparin filter, adjusted to 1 mL equivalent count.

For the 14 Mf-positive participants, the Mf counts are shown in Figure 2 for the 2018 survey and the current 2019 study at days 0, 7 and (for one participant) 30. At the 2019 visit 1, Mf were identified on filters from all 14 participants, with counts ranging from 2 to 632 per mL. The different sample and specimen types (filters or slides) were concordant (positive/negative) before treatment in 12 out of 14 (86%) persons (Figure 2). The exceptions were:

- ID-6 who was Mf-negative on the venous heparin slide but positive on all other sample and specimen types available;
- ID-8 who was Mf-positive with 2 Mf/mL on the venous filter but negative on all other sample and specimen types available.

Considering Mf densities pre-treatment in heparin fingerprick slides, Figure 2 notes the reported responses given about the 2018 MDA by each individual in 2018 and 2019. If MDA had been taken, a marked decline in the Mf density would have been expected between 2018 and 2019 pre-treatment Mf density, but a steep decline was seen only in one person who reported Yes both times (ID-8, a 5 year old child) (Figure 2). Lesser declines between 2018 and 2019 baseline were seen in some other participants reporting Yes both times, (ID-6,12 and 14). Others reporting Yes both times showed little decline (ID-3,9) or even increase (ID-5,13). Responses for those consistently reporting No (ID-2,4,10) or changing report from Yes to No (ID-1,7,11) showed a range of patterns from 2018 to 2019 baseline but no steep declines.

One person (ID-11) experienced adverse reactions to the MDA medications, withdrew from the study at visit 2 (3 h post-treatment) and declined to provide further blood samples. The participant felt nauseous, vomited and had a headache in the hours following taking the IDA. They had improved by the following day. They did not seek medical care, did not require hospitalization, or as far as we are aware required any treatment.

Of the 13 participants who agreed to provide a post-treatment blood sample at visit 3, 12 were cleared of detectable Mf on all their 7-day post-treatment blood samples and specimen types examined (Figure 2). One person (ID-1) had persistent Mf at day 7 (at a reduced density) in one filter (EDTA) and one slide (venous heparin), but was negative on heparin filter and heparin fingerprick slide. They had no detectable Mf in any sample at day 30 (no additional IDA doses were given) (Figure 2).

Mf counts on fingerprick slides in 2018 for the 4 Mf positive persons who were not followed up in 2019 ranged from 18 to 959 per ml, i.e., their counts in 2018 were within the range of the followed-up participants.

### 3.5. Pharmacokinetics

Considering that we observed Mf clearance in 12 of 13 (92.3%) participants by day 7 and in the additional participant by day 30, pharmacokinetic studies were deemed to be not necessary and not conducted.

### 3.6. Comparions of Mf Density in Different Sample and Specimen Types

The differences between counts observed in different heparin sample and specimen types were compared and the results given in the Supplementary Materials (Table S1 and Figure S3). Mean and median counts were significantly higher in venous filter samples than in venous or fingerprick slide samples (Table S2) but not different between the two types of fingerprick specimens. Correlations between log (Mf/mL + 1) counts ranged from 0.529 to 0.729, and were significant between venous filters and both specimen slide types (venous and fingerprick) (Table S2).

At baseline pre-treatment in 2019 we had only 4 participants with EDTA samples taken (one of whom withdrew after day 0) and thus a comparison of densities between heparin and EDTA could not be done. Nevertheless, it was noted that Mf densities detected in EDTA blood tended to be lower than with heparin (ID-11, 12 and 13) although ID-14 was an exception for the venous EDTA filter which was similar to the venous heparin filter (Figure 2).

## 4. Discussion

IDA was highly effective for clearing Mf in Samoa when medications were taken while directly observed, and using the recommended weight-based dosages. Mf were cleared completely by day 7 after treatment in 12 of 13 participants followed-up, and by day 30 in the remaining participant. Overall, this study found that persistence of Mf in a proportion of individuals after the triple-drug MDA programme in 2018 was unlikely to have been due to drug resistance in the local filarial worm population. More likely explanations include inadequate dosage due to incorrect weighing, administration of insufficient numbers of pills, and/or flawed reporting about participation, i.e., non-compliance or partial compliance in taking pills. Thus future MDAs must provide accurate weighing scales, as well as thorough training for MDA drug distributors in correct dosage by weight. The practice of directly observed treatment must be emphasized in future MDAs.

Our results showed that reported MDA taking may not be accurate. Based on the findings of full clearance of Mf after observed correct treatment doses in the current study, it seems unlikely that the seven participants who consistently reported Yes to the 2018 MDA when asked in both 2018 and 2019 actually took the full correct dose. This may be an error in the dose provided, or failure to swallow all the pills. It may also be due to social desirability bias (participants respond with the ‘correct’ answer that they think surveyors want to hear, especially in front of family members), or incorrect proxy reporting for children or absent family members. In Samoa, surveys tend to be conducted in family groups, often with little privacy during questionnaires. Persistence of Mf positivity after MDA is more understandable in those who consistently reported No in both surveys (3 of 14) or changed answers from Yes to No or Incomplete (4 of 14 participants).

Incorrect reporting of MDA participation as Yes in up to half of the Mf positive participants may not be as grave a problem as it initially appears however. The participants represent a biased sample of the survey population since they were identified and selected due to being Mf-positive (presumably because they were more likely not to have taken MDA). Reported MDA compliance in family members did not change between surveys to a similar extent, and their compliance in both years (88.9%) was no different than that reported in the full survey [17]. Nevertheless, the findings show that it is important to ask survey questions in a manner most likely to elicit an accurate response. For example, interviewing people individually rather than in front of their families, avoiding proxy responses from one person for a whole family, or stressing that the there is no ‘right’ answer, and probing further if answers seem rushed. When MDA is given to some children at school, as it was in Samoa, parents or guardians may be unaware of their children’s participation or not, and ‘don’t know’ answers should be acceptable.

Regarding the laboratory methods, filters sample a much larger volume of blood than slides and were better for assessing Mf presence and density. However, the method is time consuming, provides greater exposure of lab workers to blood and potential aerosols, and is difficult to apply on a large scale. Slides made from 60 uL of either venous or fingerprick blood were acceptable for determining Mf clearance, but using slides exclusively would have missed some Mf-positive participants. All participants (*n* = 14) were still Mf-positive by venous blood on filters before treatment, but two participants were negative by slide from the same venous sample, and one of them was also negative on the fingerprick slide. This finding of greater sensitivity of filters for determining Mf positivity corresponds with the review of Vinkeles Melchers et al [25].

A limitation of the study is the relatively small sample size and the inability to follow up all those who were identified as Mf-positive in 2018. Additionally, at the beginning of the study we did not know the best anticoagulant, sample or specimen type to use and therefore the methods evolved during the study. We made slides from anticoagulated blood which limited our ability to compare with previous studies of Mf density which used fingerprick slides made from whole blood, either directly from the finger or collected in capillary tubes [26,27]. We also used high flow spring loaded lancets compared to the manual type used in earlier studies.

Our study was not designed to answer additional questions that may arise about the effect of IDA on adult worms or the kinetics of reappearance of Mf from adult worms after IDA. Our conclusions about lack of resistance to the drugs in Mf are an indirect measure of resistance. Further studies on kinetics of adult worm Mf production would be important to do when possible.

Mf counts had a greatly skewed distribution; counts in different sample and specimen types were compared after log transformation by both parametric and non-parametric tests, which gave consistent results. Estimated mean log and median Mf densities were significantly higher in the filtered venous blood than on slides made either from the same venous sample or concurrently from fingerprick blood. Comparisons of counts detected no significant difference in densities on slides made concurrently from venous or fingerprick blood.

Our finding of no difference in estimated density between venous and fingerprick slide samples contrasts with previous findings implying that density was higher in fingerprick than venous blood [26,27]. However, previous studies compared Mf density from venous filters with fingerprick slides made with whole blood (20 uL applied in one circular sample rather than 60 uL in 3 lines), not heparinized blood as used in this study for both venous and fingerprick slides. It is not clear why slides from venous blood samples in our study would yield lower counts than filters from the same blood samples. The explanation for both findings may be that Mf in heparinised blood do not stick as well to slides as to filters, or as well as on slides made with whole blood.

We found that EDTA appears preferable to heparin as anticoagulant for filtered blood to reduce clumping of Mf and make counting easier (with the caveat that densities in pretreatment blood tended to be lower in EDTA than heparin blood). Further studies are required to determine which anticoagulant is better for filtered blood studies, but filters are preferred over slides alone for greater sensitivity of detecting Mf and counting their density. If EDTA is used, separate samples need to be taken for FTS antigen determination if needed, since the manufacturer specifies heparinized blood. For studies with large numbers of samples where filters from venous blood are not feasible, slides made with whole blood (not included here) may be more sensitive than those made with heparinized or EDTA blood, and this should be tested given the greater emphasis being placed on Mf prevalence as elimination nears.

## 5. Conclusions

The triple-drug MDA strategy in Samoa was effective at clearing Mf by 30 days post-treatment when given and taken at the recommended weight-based dosage. Our study did not identify any evidence of drug resistance to IDA in Samoa.

## Figures and Tables

**Table 1:** Weight-based dosing schedule for triple-drug MDA in Samoa, 2018.

Weight Range (kg)	Number of Ivermectin Tablets (3 mg)	Number of DEC Tablets (100 mg)	Number of Albendazole Tablets (400 mg)	Total Number of Tablets
<15 kg (or 2–4 years old)	0	1	1	2
15–23 kg	1	1	1	3
24–38 kg	2	2	1	5
39–53 kg	3	3	1	7
54–68 kg	4	4	1	9
69–83 kg	5	5	1	11
84–98 kg	6	6	1	13
99–124 kg	7	7	1	15
>124 kg	8	8	1	17

Source: Samoa Ministry of Health. kg: kilogram; mg: milligram; DEC: diethylcarbamazine.

**Table 2:** Flowchart of follow up of Mf positive people and their household members in the Monitored Treatment study, Samoa 2019.

Household Visit	Timing	Activities	Venous Blood Samples	Fingeprick Blood Samples
1	Day 0	Enrolment of Mf-positive participants from 2018 and their household members Consent Questionnaire by interview Weigh Give directly observed treatment of IDA Note any immediate adverse events	Heparin & EDTA * samples for: Mf filters Slides Plasma	Heparin samples for: FTS Slides if positive FTS result
2	Day 0, 3 h post- treatment	Note any adverse events from IDA	Heparin & EDTA samples for: Plasma	Not collected
3	Day 7	Discuss results of FTS and slides from Day 0 Enrol any additional household members who were not present on Day 0	Heparin & EDTA samples for: Mf filters Slides Plasma	Heparin samples for: FTS Slides if positive FTS result
4	Day 30 (only visited if Mf-positive on Day 7)	Discuss results of slides from Day 7	Heparin & EDTA samples for: Mf filters Slides Plasma	Heparin samples for: Slides

* EDTA filters were done starting with participant ID 10; obtained for 4 of 14 participants at visit 1.

**Table 3:** Participant characteristics and MDA (2018) participation reported in 2018 and 2019.

Participant ID Number	Age (Years)	Gender	Reported (in 2018) Taking 2018 MDA	Reported (in 2019) Taking 2018 MDA
1	59	M	Yes	No
2	46	F	No	No
3	50	F	Yes	Yes
4	44	F	No	No
5	60	M	Yes	Yes
6	52	F	Yes	Yes
7	20	F	Yes	No
8	5	F	Yes	Yes
9	44	M	Yes	Incomplete ^1^
10	64	F	No	No
11	34	F	Yes	No
12	74	M	Yes	Yes
13	58	M	Yes	Yes
14	50	M	Yes	Yes
No blood samples: interview only or missing in 2019				
15	27	M	Yes	Unknown
16	73	M	Yes	No
17	41	F	No	No
18	31	F	Yes	No

^1^ Reported that they were given only two types of pills in 2018, and not weighed using scales.

**Table 4:** MDA participation in 2018, reported in 2018 and 2019.

			MDA Participation in 2018			
Group	Status	*n*	Reported in 2018		Reported in 2019	
Mf-positive participants identified in 2018	Ag pos + Mf pos	14	Yes	11	Yes *	8
			No	3	No	6
Family members	Ag pos + Mf neg	5	Yes	4	Yes	4
			No	1	No	1
	Ag neg + Mf neg	22	Yes	20	Yes	20
			No	2	No	2
	All family members	27	Yes	24	Yes	24
			No	3	No	3

Mf: microfilaria; Ag: antigen; pos: positive; neg: negative. * Includes the participant who reported taking only 2 of the three MDA drugs.

## Data Availability

All relevant data is included in the paper.

## Supplementary Materials

The following are available online at https://www.mdpi.com/article/10.3390/tropicalmed6020044/s1. The Supplementary File includes Figures S1–S4 and Tables S1 and S2 with explanatory text.
