# Supplementary material for: Triple-Drug Treatment Is Effective for Lymphatic Filariasis Microfilaria Clearance in Samoa
Source: Trop Med Infect Dis. 2021 Apr 1;6(2):44. doi: 10.3390/tropicalmed6020044 (PMC8167748; doi:10.3390/tropicalmed6020044)
Supplement: Supplementary file 1 [file tropicalmed-06-00044-s001.pdf]

Article

Triple-Drug Treatment Is Effective for Lymphatic Filariasis Microfilaria Clearance in Samoa

Patricia M. Graves [1,*], Sarah Sheridan [2], Jessica Scott [1], Filipina Amosa-Lei Sam [3], Take Naseri [4], Robert Thomsen [4], Christopher L. King [5] and Colleen L. Lau [6,7]

Supplementary Materials

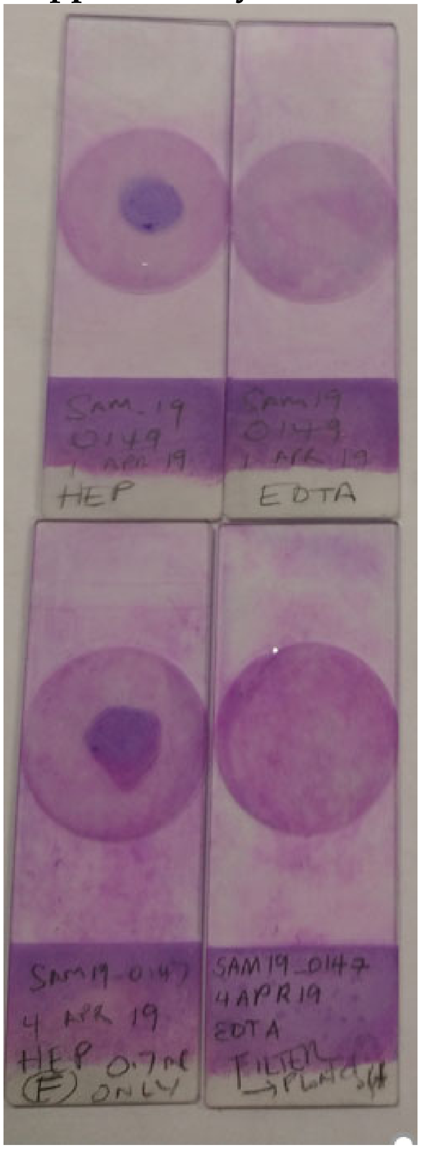

Figure 1. Comparison of filters made from heparin or EDTA blood of two participants (ID number 11, 34 year old female; ID number 12, 74 year old male; 2019 day 0. Heparin on left and EDTA on right.

A: ID number 10, 64 yr old female, ×100 (also shows fibre artefact on filter)

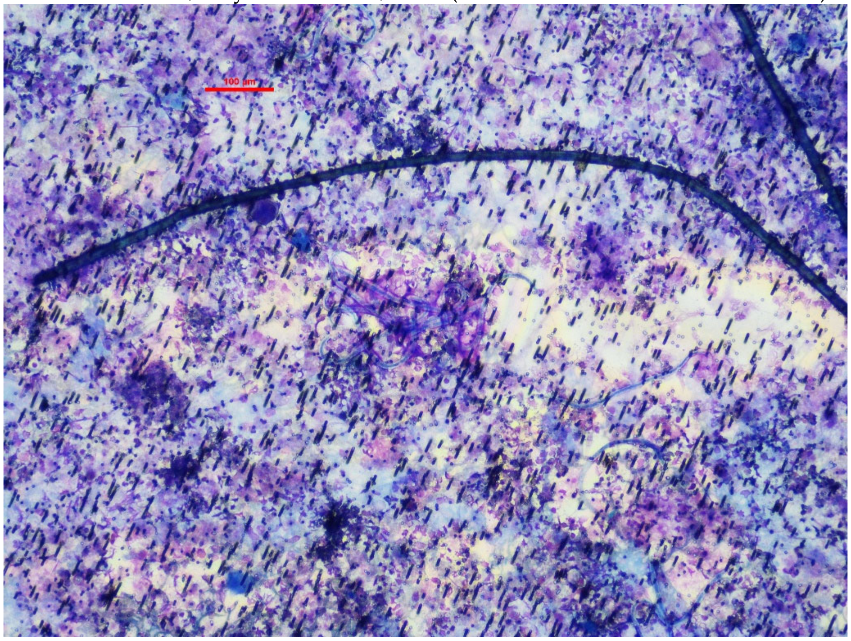

B: ID number 10, 64 yr old female, ×400

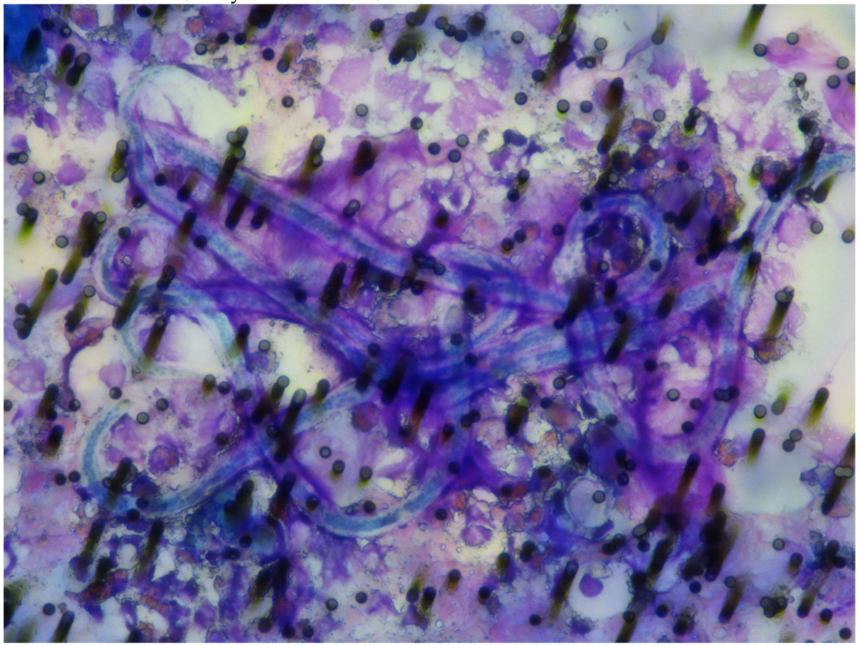

Figure S2. Clumping within the central higher density region on filters made with venous heparinized blood.

Mf Density by Sample Type and Specimen Examined

For each person at day 0, visit 1 (before treatment), we compared the median and log Mf density per mL+1 for concurrent samples of three sample/specimen types. The three groups of sample and specimen types were as follows: VF: venous blood filters, VS: venous blood slides, FPS: fingerprick blood slides. Only heparinized samples were evaluated, since only 2 of 14 particpants had EDTA blood at this time point.

Mean counts were highly skewed and subject to bias by outlying high counts (e.g. for participant 1, fingerprick slide Mf density of 3467/mL (see Figure 3 in main paper), so mean log (Mf/mL+1) counts were used. Summary measures for the different sample and specimen types are shown in Table S1. Median values and percentiles are shown in Figure S3.

Table S1: Mf counts on heparinized blood from pre-treatment samples, visit 1 at day 0.

	N	Mean Mf/mL (SD)	Range	Mean log (Mf/mL+1) (SD)	Median Mf/mL
Venous filters (VF)	14	227.9 (217.1)	2 to 634	4.5 (1.8)	237.8
Venous slides (VS)	14	286.9 (334.2)	0 to 875	1.9 (1.0)	150.0
Fingerprick slides (FPS)	14	457.3 (900.4)	0 to 3467	2.1 (0.9)	154.5

Mf: microfilaria; mL: millilitre; SD: standard deviation

Significant differences were observed between mean log in different sample and specimen types. Mean log (Mf/mL+1) count was highest on the VF, and lower on VS and FPS (Table S1). Comparison of mean logs took account of the non-independence of the three sample and specimen types from the same individuals, and was adjusted for non-sphericity in the analysis. Analysis of variance for between versus within groups sum of squares, adjusting for non-independence and mulitple comparisons, showed there was a highly signficant difference between the mean log (Mf/mL+1) counts from the different sample/specimen types (VF, VS and FS) ($F=18.68$, $df=2$, $p<0.001$).

Median counts were highest in the VF and lower but similar to each other in the two slide groups (VS and FPS) (Table S1 and Figure S3). Overall the median Mf/mL were significantly different between groups by non-parametric K sample test on the equality of medians ($Chi^2 = 6.86$, $p = 0.032$). However this test does not take into account the non-independence of the samples.

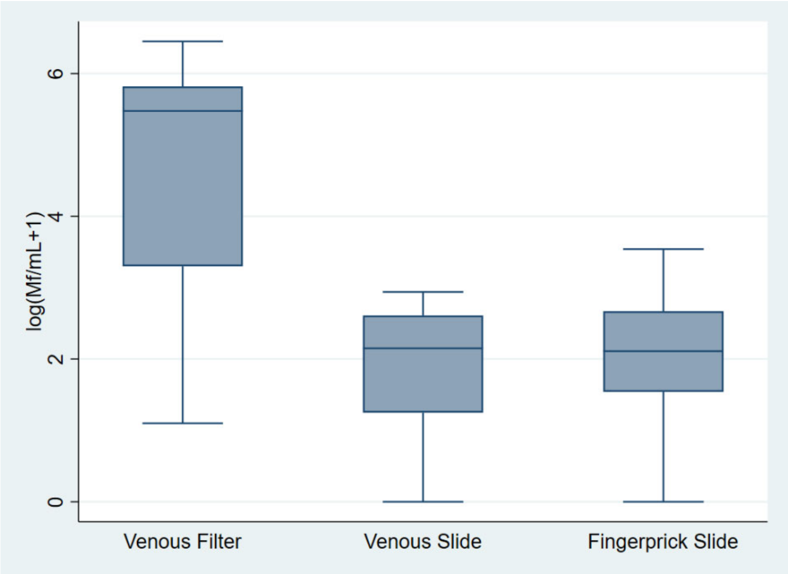

Figure S3: Median and percentiles of log (Mf/mL+1) in different sample and specimen types on day 0 (pre-treatment). Mf: microfilaria; mL: millilitre.

Pairwise comparisons by ANOVA in mean log (Mf/mL+1) counts for three groups are given in Table S2. Significant differences were observed between the filter group VF and both VS and FPS slide groups, but not between the two slide groups (VS and FPS).

These relative differences in mean logs by group were also assessed by non-parametric Wilcoxon matched-pair sign rank test. Pairwise non-parametric comparisons given in Table S2 showed no difference between log venous and fingerprick slide counts, but significantly higher counts in the venous filter group compared to either of the others.

Table S2: Comparison of log (Mf/mL+1) counts in heparinized blood from pre-treatment samples, visit 1 at day 0.

Comparison	Pairwise ANOVA		Wilcoxon Matched Pairs Signed Rank Test		Correlation Coefficient	
	**Difference in mean logs (95% CI)**	**P**	**z**	**P**	**r**	**P**
VF versus VS	2.63<br>(1.86 to 3.40)	<0.001	3.23	<0.001	0.667	0.009
VS versus FPS	-0.15<br>(-0.52 to 0.21)	0.641	-0.57	0.604	0.529	0.052
VF versus FPS	2.47<br>(1.60 to 3.34)	<0.001	3.17	<0.001	0.779	0.001

VF: venous filter; VS: venous slide; FPS: fingerprick slide; ANOVA: analysis of variance; Mf: microfilaria.

There were correlations between the log (Mf/mL+1) counts by individual for all three sample and specimen types (Table S2), but this was weakest for VS versus FPS. Graphs of log (Mf/mL+1) individual counts for are shown in Figure S4A: VF versus VS; Figure S4B: VS versus FPS; and Figure S4C: VS versus FPS.

A:

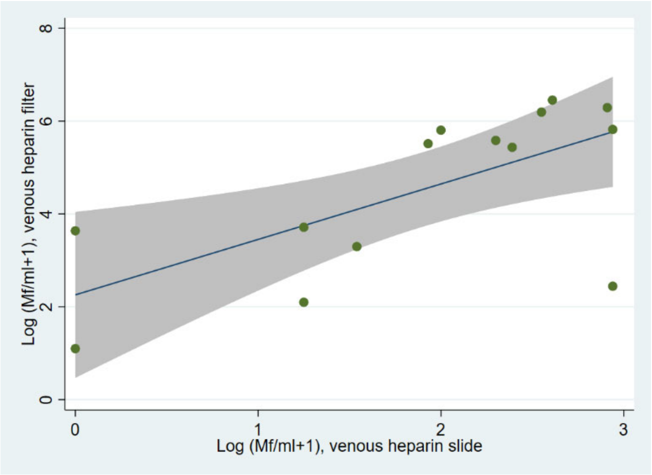

B:

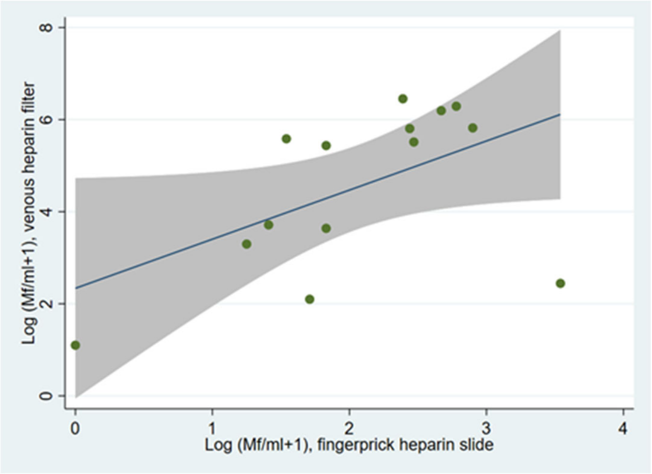

C:

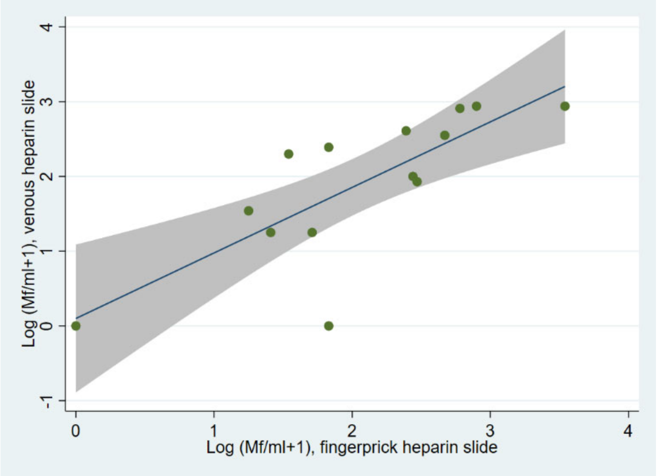

Figure S4: Mf density (log (Mf/mL+1)) in heparinized blood samples at day 0 pre-treatment. A: venous filter versus venous slide; B: Venous slide versus fingerprick slide; C: venous filters versus fingerprick slide.
